# T4‐like myovirus community shaped by dispersal and deterministic processes in the South China Sea

**DOI:** 10.1111/1462-2920.15290

**Published:** 2020-11-03

**Authors:** Huifang Li, Lu Liu, Yu Wang, Lanlan Cai, Maoqiu He, Long Wang, Chen Hu, Nianzhi Jiao, Rui Zhang

**Affiliations:** ^1^ State Key Laboratory of Marine Environmental Science College of Ocean and Earth Sciences, Fujian Key Laboratory of Marine Carbon Sequestration, Xiamen University (Xiang'an) Xiamen Fujian China; ^2^ Department of Ocean Science The Hong Kong University of Science and Technology Hong Kong China; ^3^ State Key Laboratory of Trophic Oceanography, South China Sea Institute of Oceanology Chinese Academy of Sciences Guangzhou China

## Abstract

As the most abundant and genetically diverse biological entities, viruses significantly influence ecological, biogeographical and evolutionary processes in the ocean. However, the biogeography of marine viruses and the drivers shaping viral community are unclear. Here, the biogeographic patterns of T4‐like viruses and the relative impacts of deterministic (environmental selection) and dispersal (spatial distance) processes were investigated in the northern South China Sea. The dominant viral operational taxonomic units were affiliated with previously defined Marine, Estuary, Lake and Paddy Groups. A clear viral biogeographic pattern was observed along the environmental gradient from the estuary to open sea. Marine Groups I and IV had a wide geographical distribution, whereas Marine Groups II, III and V were abundant in lower‐salinity continental or eutrophic environments. A significant distance‐decay pattern was noted for the T4‐like viral community, especially for those infecting cyanobacteria. Both deterministic and dispersal processes influenced viral community assembly, although environmental selection (e.g. temperature, salinity, bacterial abundance and community, etc.) had a greater impact than spatial distance. Network analysis confirmed the strong association between viral and bacterial community composition, and suggested a diverse ecological relationship (e.g. lysis, co‐infection or mutualistic) between and within viruses and their potential bacterial hosts.

## Introduction

Viruses are the most abundant and diverse entities in marine ecosystems and considerably impact ecological, biogeographical and evolutionary processes in the ocean. Viruses could be responsible for reducing primary production in the oceans by up to 78%, and for an important percentage (~20%) of the mortality of marine heterotrophic bacteria (Suttle *et al*., 1990; Suttle, 1994). Therefore, it is becoming increasingly clear that we need to incorporate viruses and virus‐mediated processes into our perception of marine ecology and oceanography (Suttle, 2007). Understanding the occurrence and mechanisms of the biogeographic patterns of marine viruses will help in predicting their influence on host populations and microbial community composition, and thus on large‐scale oceanic processes (Stegen *et al*., 2012; Marston *et al*., 2013; Chow and Suttle, 2015; Gregory *et al*., 2019).

Recently, researchers have observed the existence of biogeographic patterns, often defined as the presence of spatial patterns of biodiversity resulting from deterministic (selection) and dispersal (spatial distance) processes, among a wide range of microorganisms (Hanson *et al*., 2012; Chow and Suttle, 2015). Selection varies the relative abundance of microbial species based on their ability to survive and reproduce. Potential variables influencing selection include the physical, chemical and biotic factors of a given environment. Dispersal refers to the movement, and subsequent successful establishment, of a microbial species to a new location (Hanson *et al*., 2012). Williamson *et al*. (2008) implicated that marine viral communities display biogeographic patterns that are widely dispersed and influenced by local environmental selective pressures. Several other surveys also found a significant correlation between viral community structure and environmental parameters (Desnues *et al*., 2008; Marston *et al*., 2013). For instance, Angly *et al*. (2006) showed that salinity, temperature and oxygen concentration shape viral communities in the Gulf of Mexico, the Sargasso Sea and the Arctic Ocean. Frederickson *et al*. (2003) found that the physical structure of the water column influenced the structure of cyanophage communities in inlets in Canada. Studies on the influence of microbes on viral diversity and abundance both in coastal waters and the open ocean have shown that viral community structure may be strongly influenced by their hosts' communities (Fuhrman, 1999; Short and Suttle, 2002; Winter *et al*., 2005; Sandaa *et al*., 2007; Suttle, 2007; Parada *et al*., 2008; Short *et al*., 2011; Breitbart, 2012; Chow and Fuhrman, 2012; De Corte *et al*., 2016). While several studies have suggested that viral community diversity is mainly influenced by dispersal processes (Snyder *et al*., 2007; Winter *et al*., 2013), yet the influence of abiotic environmental variables, potential host community and spatial parameters are seldom investigated simultaneously in viral biogeographic studies.

The South China Sea (SCS), which is connected to the Pacific Ocean by the Luzon Strait, is one of the largest semi‐enclosed marginal seas in the world. It has a wide continental shelf in the northwest area, which receives high volumes of runoff (3 × 10^11^ m^3^ v^−1^) from the Pearl River, as well as a 4700‐m‐deep basin (Shaw and Chao, 1994; Zhang *et al*., 1999; Chen *et al*., 2001). The northern South China Sea (nSCS) is separated into three distinct regions based on bathymetric features: estuarine coastal plume (mostly eutrophic coastal region), continental shelf (transitional region) and open ocean (oligotrophic region) (He *et al*., 2009). Environmental variables such as salinity and nutrient availability often form a gradient in estuarine environments, producing a distance effect on spatial changes in microbial composition. The SCS is thus an ideal environment for investigating how viral community composition (VCC) and its control mechanisms alter with regard to distance.

T4‐like phages are a subset of tailed phages that make up a significant fraction of the viral communities (Ackermann and Krisch, 1997; Breitbart *et al*., 2002; Brum *et al*., 2013). Here, we investigated the impact of environmental selection and dispersal processes on T4‐like viral community assembly along an ~500‐km transect with an environmental gradient from the estuary to open sea. Because most viruses in the ocean infect prokaryotes (Weinbauer, 2004; Baudoux *et al*., 2007; Payet and Suttle, 2008), we hypothesized that bacterial community composition (BCC) acts as an important environmental selection parameter in shaping VCC. To test this hypothesis, we used high‐throughput sequencing to examine the composition and diversity of T4‐like viruses and their potential bacterial hosts in the northern SCS.

## Results

Samples were collected in the current study from the nSCS along a gradient extending from an estuarine environment to the continental shelf and on to the open sea (Fig. S1). Overall, three clusters of water samples were recognized based on temperature and salinity (Fig. S2, Table S1). Cluster 1 (sample A9‐5 m) could be identified as the estuarine water mass (EWM) with relatively high temperature (28.1°C) and low salinity (31.19), whereas cluster 3 (comprising samples J2‐50 m, I1‐75 m, K3‐75 m, K4‐75 m and SEATS‐75 m) could be identified as the sub‐surface open sea water mass (ssOWM) and had the lowest water temperature (21.9–23.8°C) and highest salinity (34.22–34.36) among all samples. Cluster 2 samples showed a wider range of salinities (33.04–33.70) and temperatures (28.6–30.0°C) and could be separated into two sub‐clusters based on trophic features. The first sub‐cluster contained samples with a salinity <33.12 (samples J1‐5 m, J2‐5 m and J3‐5 m), representative of the continental water mass (CWM; transitional region between eutrophic and oligotrophic zones), while the second sub‐cluster consisted of samples from the surface open sea water mass (sOWM; oligotrophic region; samples J4‐5 m, J4‐25 m, I1‐5 m, D‐5 m, K2‐5 m, K3‐5 m, K4‐5 m, and SEATS‐5 m), which showed higher salinity (33.35–33.70).

The abundance of heterotrophic bacteria was highest in the eutrophic EWM sample, followed by the continental and open sea environment samples (Table S1). Among the autotrophic cyanobacteria, the highest concentration of *Prochlorococcus* was observed in the sOWM samples, followed by the ssOWM and costal CWM and EWM samples. *Synechococcus* were most abundant in samples from the continental and open sea environments but were less abundant than *Prochlorococcus* in the sOWM samples (Table S1). In addition, the abundance of picoeukaryotes was higher in ssOWM and EWM samples (Table S1).

### Diversity among viral and bacterial communities

In total, 122 969 viral sequences and 750 658 bacterial sequences were generated after quality control in this study. We recovered 824 viral operational taxonomic units (vOTUs) and 1111 bacterial OTUs (bOTUs). Good's coverage values from T4‐like viral and bacterial sequences varied from 96.3% to 99.0% and 99.7% to 99.8% (Table S2) respectively. The rarefaction curve generated from the sequence data approached saturation (Fig. S3). Those indicated that the diversity of T4‐like viruses and bacteria in the library was most covered. A clear inverse correlation was displayed between species richness (number of OTUs) and frequency of occurrences for both vOTUs and bOTUs (Fig. S4). The majority of the vOTUs and bOTUs appeared in only a few samples, with few OTUs occurring widely across samples. Moreover, there was a positive relationship between the average contribution of an OTU to the total community and its occurrence (Fig. S4). For example, vOTU and bOTU with the highest average contribution (12.6% and 6.28% of the community respectively) were observed in all samples. The rank‐abundance plot of the average contribution per OTU had a clear inverted exponential curve (Fig. S4B and D), showing that the viral and bacterial community structures were uneven.

### Phylogeny of T4‐like viruses and bacteria

**Fig. 1:**
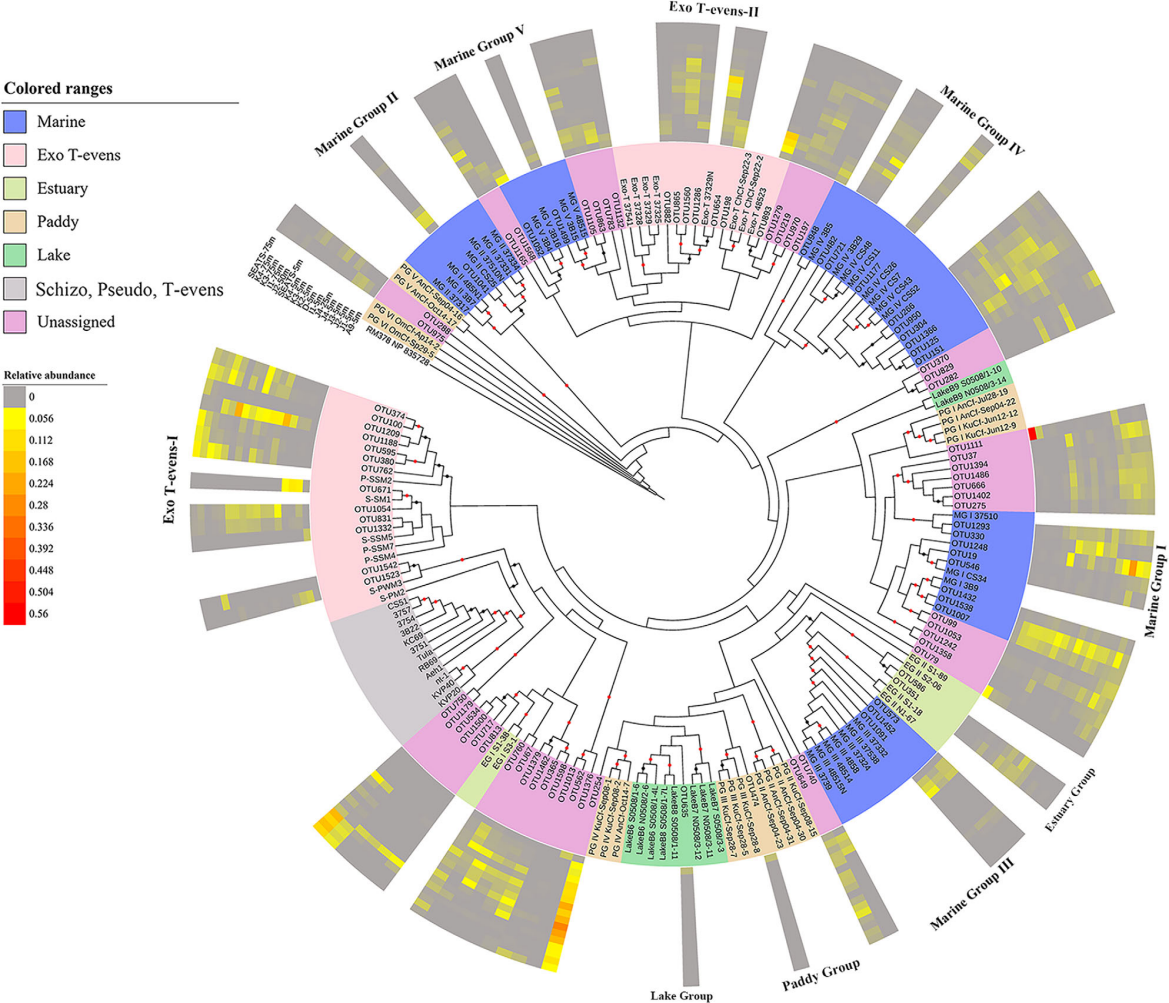
Maximum‐likelihood phylogenetic analysis based on amino acid sequence of the obtained *g23* OTUs. OTUs with a relative abundance of >1% in each sample (a total of 93 OTUs) from the nSCS were selected. Black and red dots show internal nodes with >50% and >80% bootstrap (1000‐fold replicated) support respectively. Different coloured ranges indicate *g23* sequences from different groups or origins. The outer coloured rings indicate the relative abundance of each OTU in each sample. Light grey indicates sequences not detected in the samples (relative abundance of 0%). [Color figure can be viewed at wileyonlinelibrary.com]

**Fig. 2:**
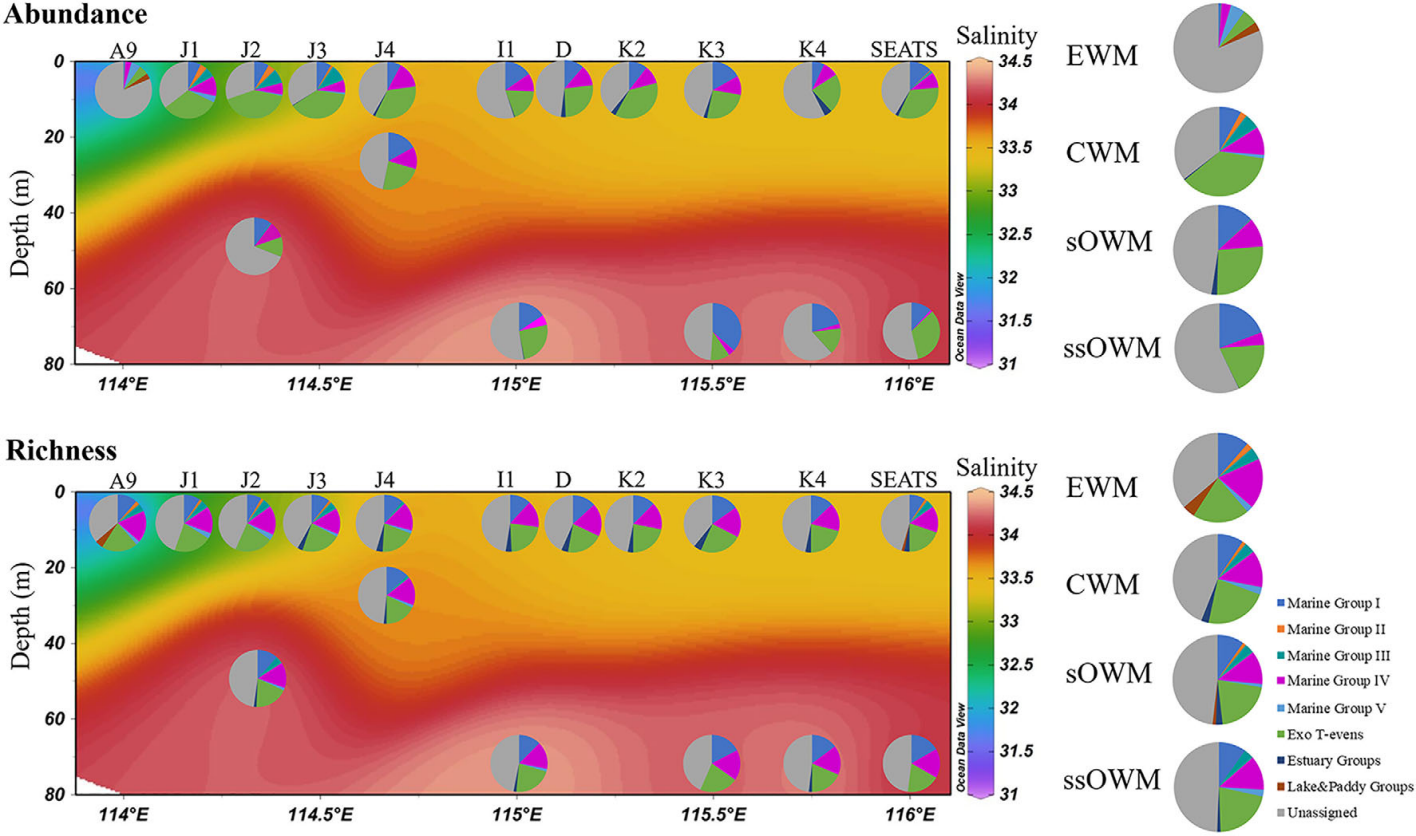
Comparison of the relative distribution of each group of T4‐like viruses obtained from the transect of the nSCS. [Color figure can be viewed at wileyonlinelibrary.com]

Basic local alignment search tool analysis of nucleotide sequences indicated that the majority of the dominant vOTUs (relative abundance >1%, containing 93 OTUs and 37 900 sequences) in the nSCS samples were highly similar to those uncultured T4‐like viruses. The sequences were affiliated with previously defined Marine Group, Estuary Group, Lake Group and Paddy Group T4‐like viruses (Fig. 1). The most abundant myovirus genotypes were Marine Groups, accounting for 47.31% of richness and 49.06% of abundance (Fig. 2). The Marine Group can be subdivided into Marine Groups I–V, which are usually recovered from environmental sequences, and the Exo T‐evens, which infect cyanobacteria (Filée *et al*., 2005). Exo T‐evens group was the most diverse and abundant subgroup in the current study (25.30% of abundance, 21.50% of richness), and could be divided into two clusters. The Exo T‐evens‐I sequences were mainly similar to *g23* sequences from *Prochlorococcus* phages (e.g. P‐SSM4 and P‐SSM2) and *Synechococcus* phages (e.g. S‐SSM5 and S‐SM1), whereas the Exo T‐evens‐II sequences were most similar to environmental viral *g23* sequences mostly recovered from the Pacific Ocean. Sequences belonging to Marine Groups I (8 OTUs, 12.96% of sequences) and IV (10 OTUs, 8.27% of sequences) were recovered from all four water masses in the nSCS. Spatially, Marine Group IV was most abundant in samples from the CWM and sOWM, while Marine group I was most prevalent in the sOWM and ssOWM samples (Fig. 2). In addition, Marine Groups II and III were mainly present in the CWM samples, while Marine Group V was mostly recovered from the EWM (Fig. 2). In the nSCS, 0.98% of *g23* sequence (2.15% of richness) fell within the Estuary Groups, while 0.19% of sequence (2.15% of richness) were affiliated with the Paddy and Lake Groups (Fig. 2). None of the sequences were grouped with phages belonging to the T‐even and Pseudo T‐even groups, whose hosts primarily inhabit animal guts and are considered essentially absent from the open sea (Fuhrman *et al*., 1993; Bano *et al*., 2004), despite being observed in the marine environment in previous studies (Filée *et al*., 2005).

In total, 17 bacterial phyla were identified across all samples. *Proteobacteria* was the most abundant phylum across all stations with the exception of sample J2‐5m. *Bacteroidetes* and *Cyanobacteria* were highly abundant in samples from the open sea (Fig. S5A). At the class level, *α‐* and *γ‐Proteobacteria* and *Bacteroidia* were highly abundant in all samples. *β‐Proteobacteria* were more abundant in samples from the estuarine environment than in any other samples. Samples collected from the open sea had a high abundance of *Oxyphotobacteria*, *Rhodothermia* and *Acidimicrobiia* (Fig. S5B), while *Phycisphaerae*, *Actinobacteria* and *Oxyphotobacteria* were enriched in CWM and EWM samples.

### Geographic patterns

**Fig. 3:**
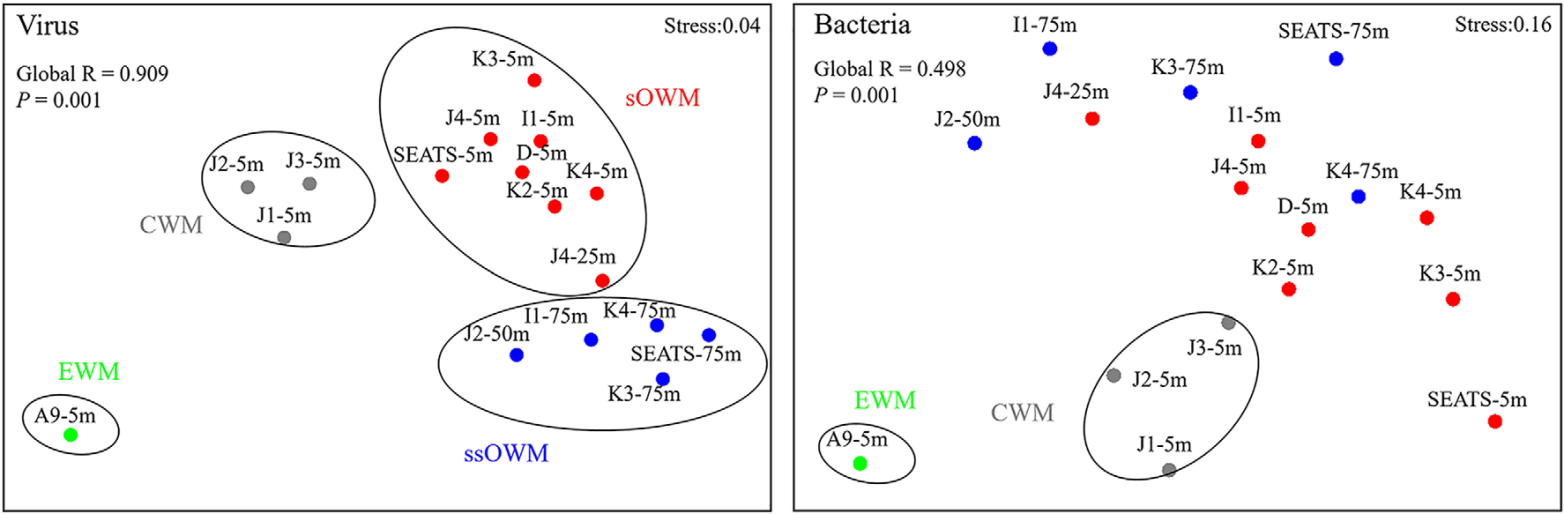
NMDS of T4‐like virus and bacteria in the nSCS based on Bray–Curtis similarity. Different oceanic water masses were marked with green (EWM), grey (CWM), red (sOWM) and blue (ssOWM) spots. [Color figure can be viewed at wileyonlinelibrary.com]

**Fig. 4:**
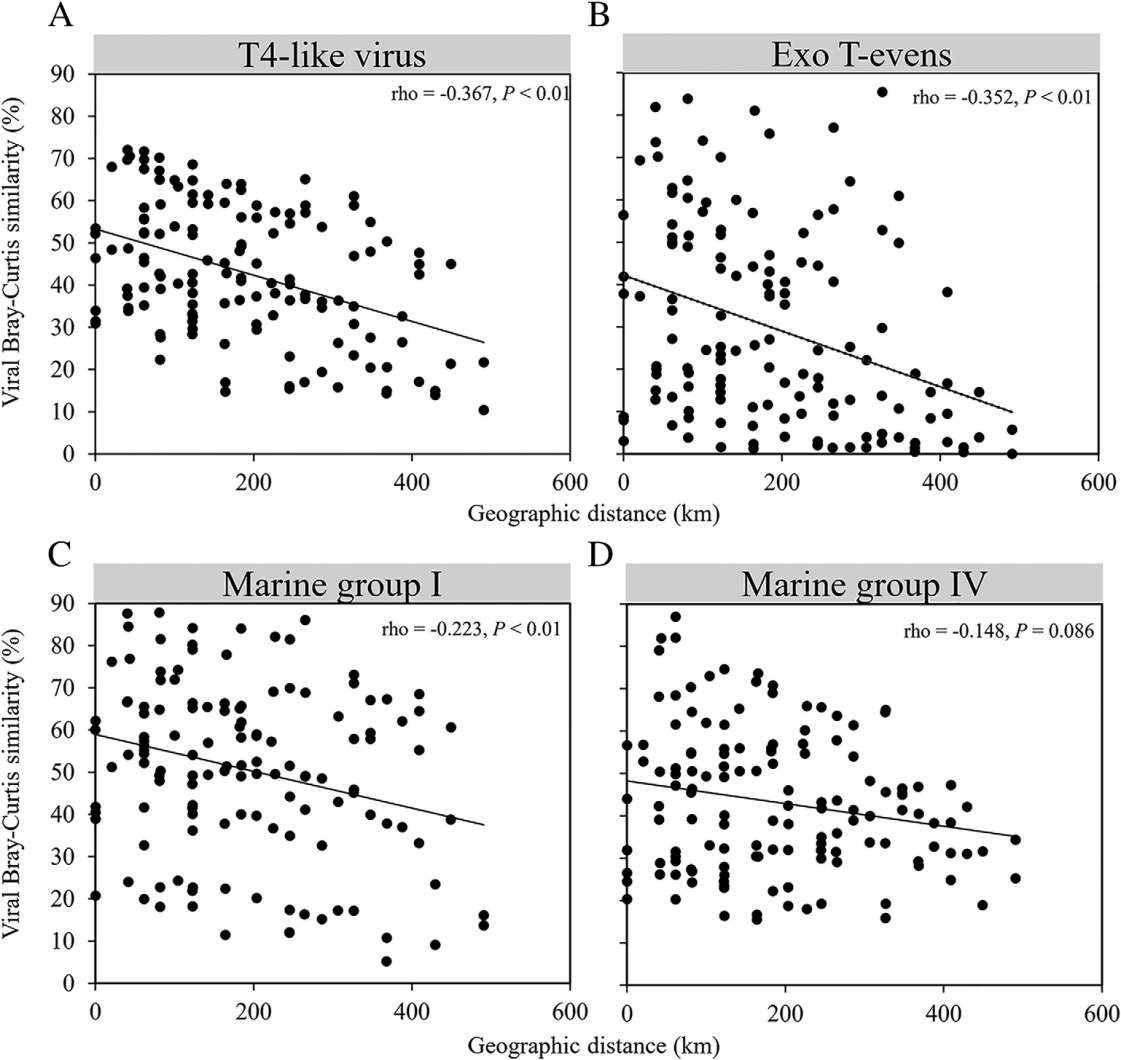
Distance‐decay patterns of T4‐like viral community (A) and major viral groups (B–D). Spearman's correlation (rho) and the significance (*P*) values are indicated.

There was an obvious separation of viral communities among the samples, with community distribution corresponding to the water mass clusters (Fig. 3, Fig. S2). This finding was supported by the analysis of similarity (ANOSIM) global test (Global *R* = 0.909, *P* = 0.001). In addition, a vertical stratification between surface (sOWM) and sub‐surface (ssOWM) communities was observed (Fig. 3). ANOSIM analysis showed that sample pairings CWM and sOWM, CWM and ssOWM and sOWM and ssOWM exhibited significant differences in viral community structure (*P* < 0.05) (Table 1). Furthermore, our results revealed a clear distance‐decay pattern between viral community compositional similarity and geographic distance (decreased community similarity with increasing geographic distance) (Fig. 4A). This pattern was observed for the most abundant T4‐like viral groups, i.e. Exo T‐evens, Marine Group I and Marine Group IV (Fig. 4B–D). Exo T‐evens and Marine Group I showed a significant relationship between community similarity and geographic distance, with the Exo T‐evens group exhibiting the highest correlation, indicative of the strongest distance‐decay pattern. Moreover, the Exo T‐evens group made the greatest contribution to the dissimilarity between the CWM and sOWM, CWM and ssOWM and sOWM and ssOWM communities. Marine Groups I and IV were mainly responsible for the dissimilarity between the sOWM and ssOWM communities, as well as between the CWM and sOWM communities, while Marine Group IV made a smaller contribution to the dissimilarity between the EWM and CWM communities (Fig. S6).

**Table 1:** Community comparison of T4‐like virus and bacteria based on ANOSIM analysis with 999 permutations.

		EWM	CWM	sOWM	ssOWM
Virus	EWM	‐	‐	‐	‐
	CWM	1	‐	‐	‐
	sOWM	1	0.987**	‐	‐
	ssOWM	1	1*	0.775**	‐
Bacteria	EWM	‐	‐	‐	‐
	CWM	1	‐	‐	‐
	sOWM	0.938	0.516*	‐	‐
	ssOWM	1	0.538*	0.287*	‐

*P < 0.05; **P < 0.01 and ***P < 0.001.

Bacterial community distribution was also corresponding to the water mass clusters (ANOSIM global test: global *R* = 0.498, *P* = 0.001) (Fig. 3, Fig. S2). The vertical stratification of the BCC was less obvious than that of the VCC. In addition, the composition of the CWM communities differed significantly with the sOWM communities (ANOSIM pairwise tests: *R* = 0.516, *P* < 0.05) and ssOWM communities (*R* = 0. 0.538, *P* < 0.05) (Table 1).

### Environmental selection and dispersal processes associated with the patterns of viral community

Our results showed a significant correlation between the VCC and BCC (Spearman's correlation, *P* < 0.01) (Table 2). Abiotic and abundance variables and geographic distance showed a significantly negative relationship with the T4‐like viral community and with the Exo T‐evens and Marine Group I sub‐groups (*P* < 0.01) (Fig. 4; Table 2). Abiotic and abundance variables and the BCC had similar degrees of correlation (almost equivalent *r‐*values) with the VCC, while geographic distance showed a relatively weak correlation (Table 2). Moreover, abiotic and abundance variables showed a significant correlation with the VCC of sOWM (rho = −0.481, *P* < 0.05), while geographic distance was significantly correlated with the VCC of ssOWM (rho = −0.915, *P* < 0.001) (Table 2).

**Table 2:** Spearman's correlation analysis of relationships between VCC, BCC, geographic distance and Euclidean distance of all the abiotic and abundance variables.

	Euclidean distance		Bacterial community		Geographic distance	
	rho	*P*	rho	*P*	rho	*P*
T4‐like viral community	−0.472	<0.01	0.625	<0.01	−0.367	<0.01
CWM	−0.500	0.667	0.500	0.667	0.500	0.667
sOWM	−0.481	<0.05	0.297	0.190	−0.264	0.174
ssOWM	−0.212	0.556	0.818	<0.01	−0.915	<0.01
Exo T‐evens	−0.447	<0.01	0.526	<0.01	−0.352	<0.01
Marine Group I	−0.428	<0.01	0.426	<0.01	−0.223	<0.01
Marine Group IV	−0.422	<0.01	0.332	<0.01	−0.148	0.086

**Fig. 5:**
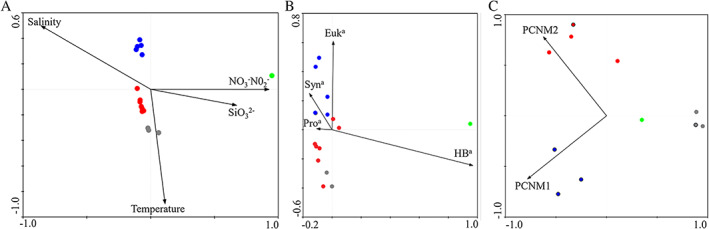
CCA was conducted to show abiotic (A) and abundance (B) and spatial (C) variables in governing the assembly of viral communities. HB^a^, Heterotrophic bacterial abundance; Pro^a^, *Prochlorococcus* abundance; Syn^a^, *Synechococcus* abundance; Euk^a^, picoeukaryotes abundance. [Color figure can be viewed at wileyonlinelibrary.com]

**Fig. 6:**
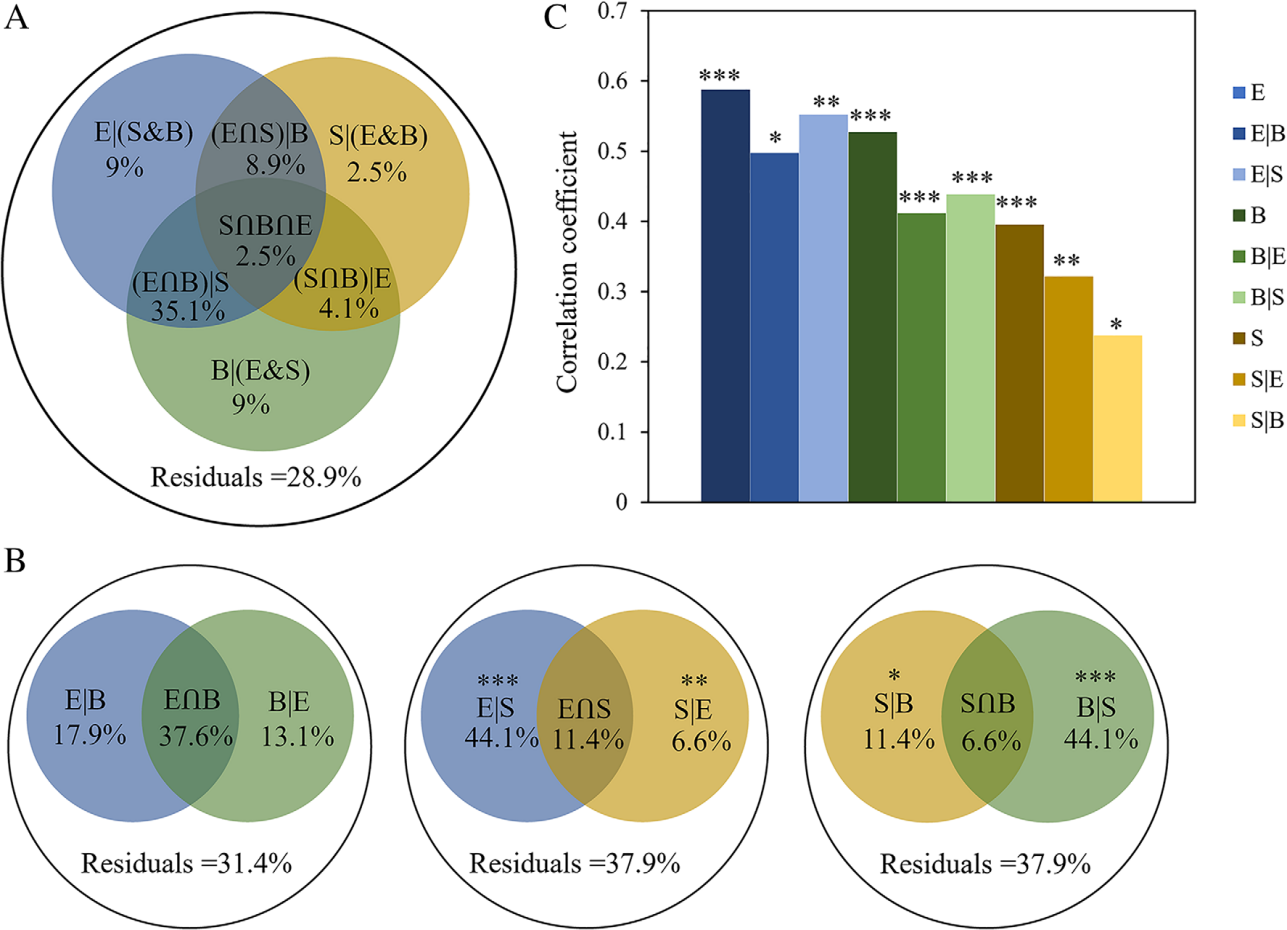
Variation in the viral community explained by abiotic and abundance (E), bacterial community (B) and spatial variables (S). (A) Variation partitioning analysis of viral community composition between bacterial community composition, abiotic and abundance and spatial variables. (B) Variation partitioning analysis of viral community composition between any two factors among bacterial community composition, abiotic and abundance and spatial variables. The variation explained by pure bacterial community composition, abiotic and abundance and spatial factors corresponds to the viral community without the effect of others by ANOVA permutation tests. *P < 0.05, **P < 0.01 and ***P < 0.001. The out circles represent 100% of the variation. (C) Mantel and partial Mantel tests to identify the correlations between the viral community structure and bacterial community composition and abiotic and abundance and spatial variables using Pearson's coefficient. ‘|’ indicates partial mantel test. *P < 0.05, **P < 0.01 and ***P < 0.001. [Color figure can be viewed at wileyonlinelibrary.com]

To further explore the mechanisms shaping the viral biogeographic patterns, the relative influence of selective factors and spatial variables in forming viral community structure were analysed. Three abiotic (temperature, salinity and NO_3_^−^ + NO_2_^−^) and one abundance (heterotrophic bacterial abundance) environmental factors and two spatial factors (PCNM 1 and 2) were shown to make a significant contribution to VCC by canonical correlation analysis (CCA) (Fig. 5). Mantel tests further indicated significant relationships (*P* < 0.05) between the above four environmental variables and the VCC (Table S3). Pure abiotic and abundance factors ((E|(S&B)), 9%) explained the same fraction of the variations as pure BCC (B|(E&S), 9.0%), and appeared to play a greater role than pure spatial factors ((S|(E&B)), 2.5%) (Fig. 6A and B). Shared environmental (abiotic and abundance and BCC) and dispersal (spatial parameters) processes (S∩B∩E) explained 2.5% of the variation in the VCC. In addition, shared abiotic and abundance parameters together with the BCC ((E∩B)|S) explained a large proportion of the variation (35.1%) in viral communities. Partial Mantel tests showed that changes in the VCC were significantly correlated with the BCC, abiotic and abundance and spatial variables in the environmental gradient from the estuary to the open sea (Fig. 6C).

### Virus–bacterium interaction

**Fig. 7:**
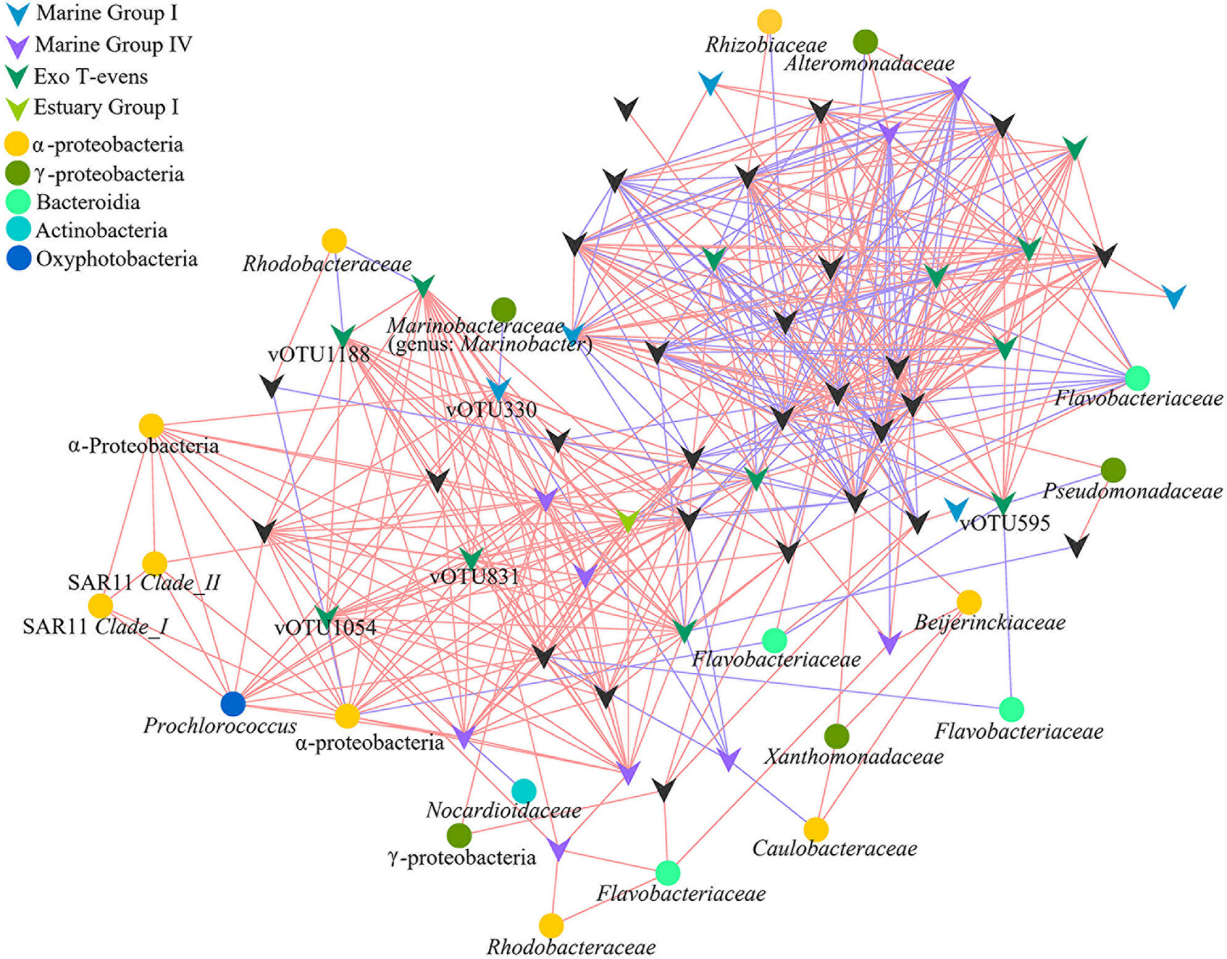
Association network comprised of dominant vOTUs and the top 10bOTUs per sample. Node degree with equal or less than two was omitted. The positive or negative linkages were determined based on Spearman's correlation analysis between any pairs of nodes. Positive linkages are shown in red, while negative linkages are shown in blue. [Color figure can be viewed at wileyonlinelibrary.com]

Co‐occurrence network based on Spearman's correlation coefficient analysis was constructed to deconvolute relationships between the predominant vOTUs and bOTUs (Fig. 7). The average path distance was 3.062, while the average clustering coefficient was 0.396. Most of the links in the network were virus‐virus associations (77.73% of 494 links), with 14.78% of the links identified as virus–bacterium. Notably, positive connections dominated the interactions between vOTUs. All of the links among the Exo T‐evens (22 links) and Marine Group I (3 links) and most of the links within Marine Group IV (8/9 links) were positive connections. There were no inter‐ and intra‐links among the Estuary, Paddy and Lake Groups.

A total of 73 links were identified between virus and bacterium, including 50 positive correlations and 23 negative correlations. Most of the linked bOTUs were identified as *α‐Proteobacteria*. In addition, there were 10 positive links between cyanobacteria and virus. A vOTU was frequently correlated to multiple bOTUs, which was often correlated with two or more vOTUs, resulting in one large interconnected cluster. There also had the correlation between one bOTU and several vOTUs. For example, one bOTU that was affiliated with the abundant family *Flavobacteriaceae* was both positively and negatively correlated with multiple vOTUs.

## Discussion

Although the spatial distribution of viruses has been investigated in some marine environments, the mechanisms underlying the development of the complicated biogeographic patterns of viral community structure are still unclear (Chow and Suttle, 2015; Le Moine Bauer *et al*., 2018). In this present study, we investigated the biogeographic patterns of T4‐like myoviruses in an ~500‐km long subtropical estuary‐sea environment and determined the relative importance of local environmental and dispersal processes on viral community assembly.

### Contrasting biogeography of the T4‐like myoviruses in the nSCS

We found a clear biogeographic distribution of T4‐like viral groups along the transect from the estuary to open sea. Both Marine Groups I and IV showed a wide geographical distribution in the nSCS, supporting previous studies showing that these groups are abundant in various marine environments, such as the Arctic glaciers, the northeastern Gulf of Mexico and the Pacific Ocean (Filée *et al*., 2005; Bellas and Anesio, 2013). Marine Group I may prefer colder water with a higher salt content, as the sequences were more abundant in the sOWM and ssOWM samples than in other samples. Marine Group III was obtained in water off the coast of British Columbia and Jericho Pier in previous study (Filée *et al*., 2005). However, our survey showed that Marine Group III was most abundant in the transitional continental shelf region than in coastal areas in the nSCS. The distribution of Marine Group II was similar to that of Marine Group III, suggesting that both sub‐groups or their hosts may be adapted to coastal shelf environments rather than the relatively high‐salinity oligotrophic open ocean. Marine Group V was mainly found in the estuary water mass samples and was rare in sOWM and ssOWM samples, suggesting that Marine Group V is well adapted to the eutrophic environments (Filée *et al*., 2005; Liu *et al*., 2017). Although the majority of the OTUs belonged to the Marine Groups, several *g23* OTUs from the nSCS clustered with Estuary, Paddy and Lake Groups (Wang *et al*., 2009; Butina *et al*., 2010; Liu *et al*., 2017). This is consistent with the findings of Sano *et al*. (2004), who showed that viruses, in particular phages, can move between different biomes (e.g. soil and seawater). The broad host range probably gives them a better chance of survival when moving between different environments (Jensen *et al*., 1998; Sano *et al*., 2004; Breitbart and Rohwer, 2005).

The T4‐like viral biogeography in the nSCS showed a clear distance‐decay pattern (Fig. 4). The higher decay rates with increasing geographic distance suggest there may be a higher dispersal limitation for viruses in the nSCS. The Exo T‐evens group was the most abundant group across all samples and showed the highest decay rate (Fig. 4), which implies the highest level of variation occurs within this group of the viral community. Despite the increased likelihood of dispersal of the highly abundant taxa, dispersal ability and establishment may also depend on the species survival strategy, the limited adaptive ability in the environment and host interactions (Östman *et al*., 2010). In the nSCS, the community composition of cyanobacteria, the major hosts of Exo T‐evens phages, showed clear spatial variation, with *Synechococcus* most common in coastal sites but *Prochlorococcus* more abundant in the open sea (Huang *et al*., 2012). This was supported by Spearman's analysis (Table 2) and the partial Mantel test (Table S4). It is worth pointing out that the strong correlation between spatial distance and the Exo T‐evens group, revealed by Spearman's analysis and Mantel test (Table 2, S4), may be attributed to the impact of spatial distance on bacterial community structure. After controlling for the bacterial community using the partial Mantel test, the significant correlation between spatial distance and the Exo T‐evens group decreased markedly (*r‐*value from 0.3687 to 0.1945) (Table S4). Therefore, we propose that the high distance‐decay rate observed for Exo T‐evens phages in our study results from the adaptive abilities of their hosts to the environment and a close interaction between phages and hosts.

### Both environmental selection and dispersal processes influenced viral communities

We found that T4‐like viral community assembly was significantly associated with local environmental factors and spatial variables (Fig. 6; Table 2). On the one hand, the environmental variables (temperature, salinity, NO_3_^−^ + NO_2_^−^, heterotrophic bacterial abundance and bacterial community structure) strongly explained the cluster patterns in the viral communities (Fig. 5; Table 2, S3). Previous analysis has shown that temperature and salinity strongly affect VCC because they play an important role in the growth and survival of viruses (Guixa‐Boixareu *et al*., 1996; Jiang *et al*., 2004; Mojica and Brussaard, 2014). Temperature can regulate infection dynamics and the sensitivity of viral lipid membranes or capsid protein, while the adsorption of viruses to host cells can be affected by salt concentration (Kukkaro and Bamford, 2009; Mojica and Brussaard, 2014). Surveys from the *Tara* Oceans expedition also demonstrated that viral community structure varied consistently with temperature and salinity on a global scale (Brum *et al*., 2013). Moreover, trophic status might be of greater importance to viral geography because viral population size and activity are highly dependent on host population size and activity (Middelboe, 2000; Suttle, 2000; Winter *et al*., 2004). This was supported by our findings showing that relatively high salinity and oligotrophic OWM and CWM samples grouped together but away from the low‐salinity and eutrophic EWM samples (Fig. 3). In addition, BCC has been suggested as to be the most important determining factor for the viral community as a result of specific virus–host interactions such as infection (Winter *et al*., 2004, 2005; Parada *et al*., 2008; Chow *et al*., 2014) (see below for detailed discussion).

On the other hand, spatial factors (dispersal processes) shape the viral community. In oceans, one of the dominant dispersal mechanisms for viruses is the movement of water masses (Frederickson *et al*., 2003; Winter *et al*., 2013; Chow and Suttle, 2015; Le Moine Bauer *et al*., 2018). Water mass transport usually contributes to a negative relationship between microbial compositional similarity and geographic distance (the distance‐decay relationship) (Green and Bohannan, 2006; Hanson *et al*., 2012). In the nSCS, spatial variables appear to contribute significantly, although to a lesser extent than environmental selection, to viral community structure. Dispersal includes establishment, meaning that at least some reproduction in the new location rather than simply just being present (Hanson *et al*., 2012). Given the small size of viruses, there are few limitations to their global dispersal, yet host availability and environmental factors may increase limitations, either directly or indirectly, that restrict geographic range (Chow and Suttle, 2015).

The partial Mantel test corroborated this result by showing that environmental selection had a great effect than dispersal processes. This result was consistent with studies conducted in the Labrador Sea (Winter *et al*., 2013). However, viral communities in the Arctic Archipelago (~1000‐km) showed a significant response to spatial factors rather than environmental variables (Winter *et al*., 2013). These differences may result from differing degrees of environmental heterogeneity and/or geographic scale (Chow and Suttle, 2015). Although samples collected in the current study covered a ~500‐km transect, the degree of environmental heterogeneity in the nSCS was higher than that in the Arctic Archipelago, meaning that large environmental gradients can increase the influence of environmental selection on viral community assembly. This observation agrees with research carried out on marine bacteria, suggesting that the relative influence of dispersal processes might shift in response to the degree of environmental heterogeneity in community assembly (Logares *et al*., 2013; Mo *et al*., 2018).

### Ecological role of the host in viral community assembly

In general, co‐occurrence correlations between microbial taxa can be explained as competition or cooperation for nutrients, material and space (Deng *et al*., 2012). In our study, co‐occurrence networks suggested complex interactions within and between viral and bacterial communities. Virus propagation is host‐dependent and infection is usually species‐ or even strain‐specific. Negative correlations between virus and bacteria indicate the presence of viral infection and lysis (Chow *et al*., 2014). Therefore, the host range of a vOTU may be suggested by negative correlations with bOTUs, with a greater number of negative connections indicative of a broad host range (such as the negative correlation between vOTU595 and three bOTUs). In the current study, bOTUs that were negatively correlated with T4‐like viruses were affiliated with *α‐* and *β‐Proteobacteria*, *Cyanobacteria*, *Bacteroidia* and *Actinobacteria*, indicating that bacteria belonging to these taxa are the likely hosts of T4‐like viruses in the nSCS. Positive correlations suggest co‐occurrence as a result of similar preferred conditions or a commensal or a mutualistic relationship between organisms cooperating within the same niche (Barberan *et al*., 2012; Faust and Raes, 2012; Chow *et al*., 2014). For example, the positive correlation between vOTU330 and *Prochlorococcus* might be a result of the viral lysis of bOTU9334 (negative correlation with vOTU330), since bOTU9334 showed 100% nucleotide sequence identity to *Marinobacter* sp. HOT4B5, which inhibits the growth of *Prochlorococcus* MIT9313 (Sher *et al*., 2011). An alternative explanation is that host lysis by viruses releases nutrients and stimulates the growth of uninfected hosts, including cyanobacteria and heterotrophic bacteria (Sher *et al*., 2011; Shelford and Suttle, 2018).

The positive associations (57.89%) within the viral community may be the result of mutualism between viruses in long‐term co‐evolutionary processes (Chow *et al*., 2014). Almost all of the intra‐links for the Exo T‐evens, Marine Group I and Marine Group IV were positive, showing that T4‐like viruses affiliated with these groups may have a co‐habitat or co‐infection (Needham *et al*., 2013; Zhang *et al*., 2017). The vOTU1054 and vOTU1188 of Exo T‐evens group, which were affiliated with *Synechococcus* phages S‐CAM8 and S‐SM2 respectively, were positively linked. Sullivan *et al*. (2003) demonstrated that phage S‐SM2, which was isolated from *Synechococcus* WH7803, has a broad host range and can infect the original host of phage S‐CAM8 (*Synechococcus* WH8017) (Sullivan *et al*., 2003). *Synechococcus* strains WH7803 and WH8017 are closely related and have similar growth conditions and ecological niches (Sullivan *et al*., 2006). These likely explain the positive link between vOTU1054 and vOTU1188 in our study. In comparison, negative correlations between viruses might indicate a competition or non‐overlapping niches between viruses or their hosts (Barberan *et al*., 2012; Faust and Raes, 2012; Chow *et al*., 2014). For example, vOTU219 and vOTU380 were affiliated with cyanophages S‐TIM4 and P‐SSM5 respectively, whose original hosts were *Synechococcus* WH8102 and *Prochlorococcus* NATL2A respectively. The negative correlation between vOTU219 and vOTU380 in our study may correspond with the different niche preference of *Synechococcus* WH8102 and *Prochlorococcus* NATL2A.

## Conclusions

The present study demonstrated the biogeographic and significant distance‐decay patterns of the T4‐like viral community in the nSCS, which is a good representative of an estuary–sea environment. We determined that T4‐like viruses belonging to Marine Groups I and IV had a wide geographical distribution, those belonging to Marine Groups II and III were mainly present in lower‐salinity continental environments, and that Marine Group V viruses preferred eutrophic coastal estuarine environments. Compared with spatial distance, environmental selection (e.g. temperature, salinity, nitrogen nutrient, bacterial abundance and community composition) had a greater impact on viral community structure. Among environmental selection factors, the bacterial community structure alone exerted almost the same degree of selective pressure as other abiotic and biotic variables in shaping T4‐like viral community assembly. In addition, network analysis suggested that BCC may impact on viral community assembly and biogeography through diverse ecological interactions, such as lysis, co‐infection and mutualism. Our data provide important ecological insights into marine viral biogeography, including community assembly mechanisms and interactions with host communities.

## Experimental procedures

### Viral and bacterial sample collection and DNA extraction

Eleven stations were visited during the cruise of the nSCS in September 2014. Water samples were retrieved from the surface (5 and 25 m) and subsurface (50 and 75 m) layers (Fig. S1). After collection, 5‐L water samples were pre‐filtered through a 20‐μm sieve and then subjected to sequential tangential‐flow filtration using a 0.22‐μm‐pore‐size filter (Millipore Corp.) to obtain bacterial concentrates (50 ml) and a 30‐kDa cartridge (Millipore Corp.) to concentrate viruses (50 ml). Total DNA was extracted from the viral concentrate using the phenol/chloroform/isoamylol method (Liu *et al*., 2017). Total DNA was extracted from the bacterial concentrate as described previously (Kan *et al*., 2008).

### Environmental parameters

Temperature, salinity and depth were measured *in situ* using conductivity‐temperature‐depth oceanic profilers (SBE9/11 plus; Sea‐Bird, USA). Temperature and salinity were used to cluster water masses using the *pamk* function of package fpc in R (Fig. S2) (R Development Core Team, 2015 (Li *et al*., 2018a). NO_3_^−^ + NO_2_^−^, SiO_3_^2−^ and PO_4_^3−^, were obtained by filtration using 0.45‐μm cellulose acetate filters and measured using a colorimetric method. To estimate the abundance of microbes, replicate samples (1.98 ml) were collected and fixed using glutaraldehyde (0.5% (vol./vol.) final concentration) at 4°C for 15 min in the dark, before being frozen in liquid nitrogen. The abundance of autotrophic picoeukaryotes, *Synechococcus* and *Prochlorococcus* was determined by direct counting without staining using flow cytometer (Accuri C6; BD Biosciences) (Jiao *et al*., 2002). Heterotrophic bacterial abundance was also determined by flow cytometer (Accuri C6) after staining with SYBR Green I (Table S1) (Marie *et al*., 1999). All data analyses were performed using FlowJo vX.0.7 software (Tree Star, USA).

### Polymerase chain reactions, sequencing and phylogenetic analysis

A fragment of the major capsid protein‐encoding gene *g23* was amplified from T4‐type phages using degenerate primers MZIA1 bis and MZIA6, as described by Filée *et al*. (2005). The amplicons were sequenced using the 454 GS FLX platform. The sequences were quality screened using MOTHUR (v.1.41.1), and chimeric sequences were discarded (Schloss *et al*., 2009; Edgar, 2010). OTUs were identified with a 97% sequence similarity. Singletons (OTUs that occurred only once) identified by *filter_otus_from_otu_table*.*py* command within QIIME were removed and samples were subsampled to 2709 (minimum sample size) from the OTU table for downstream analyses. The most prevalent *g23* OTUs, with a relative abundance >1% per sample (a total of 93 OTUs), were translated into amino acid sequences and aligned using MEGA7 (Kumar *et al*., 2016). A phylogenetic tree was constructed using the maximum likelihood method in RAxML, with 1000 bootstrap replicates (Stamatakis, 2014). The tree was viewed and graphically edited using iTOL (Letunic and Bork, 2016). Viral *g23* sequences have been deposited in the National Centre for Biotechnology Information Sequence Read Archive (NCBI SRA) database under the accession numbers SRR11786657–SRR11786673 (BioProject accession number PRJNA632563; BioSample accession numbers SAMN14912226–SAMN14912242).

The conserved V3–V4 region of the bacterial 16S rRNA gene was amplified using primers 338F and 806R (Mori *et al*., 2014; Li *et al*., 2018b). Resulting amplicons were sequenced using the Illumina MiSeq PE300 platform. Raw reads were trimmed using Trimmomatic and merged using FLASH (Magoc and Salzberg, 2011; Bolger *et al*., 2014). Thereafter, usearch61 was used to identify and remove chimeric sequences (Edgar, 2010). The remaining sequences were clustered into OTUs based on 97% sequence similarity. bOTUs with a total community abundance of >0.001% were then selected for further analysis. The number of sequences from each sample was subsampled to the same amount (30 000). Classification was carried out using MOTHUR (v.1.41.1) with SILVA (v132) reference sequences and taxonomic outline. Bacterial 16S rRNA gene sequences have been submitted to the NCBI SRA database under the accession numbers SRR11794483–SRR11794499 (BioProject accession number PRJNA632681, BioSample accession numbers SAMN14917627–SAMN14917643). Details on the primers and sequence processing of viral and bacterial sequences are supplied in the Supporting Information.

### Community diversity and structure

Rarefaction curves based on the identified vOTUs and bOTUs were estimated by PAST (v.3.18). The rarefied sequences were then used for Good's coverage and alpha diversity indices in QIIME. Non‐metric multi‐dimensional scaling (NMDS) analysis based on the Bray–Curtis similarity of VCC and BCC was performed to disentangle the difference between the samples. ANOSIM was used to test significant difference in bacterial and viral communities. Similarity percentage (SIMPER) analysis was used to conclude the contribution of major vOTUs to the observed dissimilarity between different oceanic water masses. NMDS, ANOSIM and SIMPER analyses were performed in PRIMER 6.0 (Clarke and Gorley, 2006).

### Relationships between VCC and deterministic and dispersal processes

The Bray–Curtis similarity results for VCC and BCC as well as the Euclidean distance matrices for the abiotic and abundance parameters were transformed using algorithms of log_(*x* + 1)_ in PRIMER 6.0. Geographic distance was calculated based on the coordinates of the sampling stations using a spheroidal model of Earth (Winter *et al*., 2013). The relationships among geographic distance, Euclidean distance and Bray–Curtis similarity were analysed using Spearman's rank correlations. CCA was used to connect the structure of viral communities with abiotic, abundance and spatial variables. The longest gradient lengths in the detrended correspondence analysis were >4, indicating that CCA is suitable for examining VCC. Prior to CCA analysis, the variables with a high variance inflation factor (>10) were eliminated to avoid collinearity among factors.

Variation partitioning analysis (VPA) was used to assess the relative importance of environmental and spatial parameters in forming viral community, which was widely used in ecological research to determine the relative importance of environmental selection versus dispersal processes for community structure (Legendre and Legendre, 2012; Winter *et al*., 2013; Mo *et al*., 2018). Bray–Curtis similarity is one of the most commonly used similarity quantification methods when it comes to ecological abundance data collected at different sampling locations (Legendre and Legendre, 2012; Ricotta and Podani, 2017). Principal coordinates analysis explores similarities or dissimilarities, such as Bray–Cutis similarity, and takes species identity into account, which is a better method of analysing bacterial community structure data for VPA (Mohammadi and Prasanna, 2003; Legendre and Legendre, 2012; Hamdan *et al*., 2013; Nagaraj *et al*., 2017; Hu *et al*., 2020). To avoid collinearity in the statistical calculation, principal coordinates axes with a cumulative 73.9% variation based on Bray–Curtis similarity were used to summarize the bacterial community structure (relative abundance data) for VPA. Abiotic and abundance variables, which were significantly (Mantel test, *P* < 0.05) correlated with viral community structure, were used. Spatial variables were generated using the principal coordinates of neighbour matrices analysis. The relative importance of all components was explained by pure abiotic and abundance environmental variables (E|(S&B)) (i.e. the exclusive abiotic and abundance variables excluding spatial variables and BCC), pure spatial variable (S|(E&B)), pure BCC (B|(E&S)) and the combined effects of spatial variables and BCC ((S∩B)|E) (i.e. interaction component of spatial variables and BCC excluding abiotic and abundance variables), spatial and abiotic and abundance variables ((S∩E)|B), BCC and abiotic and abundance variables ((B∩E)|S), and the combined effects of spatial, BCC and abiotic and abundance variables (S∩B∩E) respectively. The Mantel and partial Mantel tests were conducted to verify the results obtained from VPA. All these statistical analyses were performed in R.

### Network analysis

Potential interactions between viral and bacterial taxa were determined by modelling the community in network structure. Association networks were constructed using an online Molecular Ecological Network Analyses Pipeline (http://ieg4.rccc.ou.edu/MENA) (Deng *et al*., 2012). Pairwise correlation matrices were generated for the viral and bOTUs. Similarity matrices were calculated based on Spearman's rank correlation. Random matrix theory–based algorithm was used to determine the threshold of network (Deng *et al*., 2012; Zhang *et al*., 2017). Cytoscape 3.2.1 was used to describe the network, showing nodes (vOTUs and bOTUs) linked by lines that denoted positive or negative correlations.

## Supporting information


**Fig. S1.** Location of the 11 sampling stations in the nSCS. Water samples were collected from surface and subsurface layers.
**Fig. S2.** (A) Distribution of salinity and temperature among the 17 samples in the nSCS. (B) Three clusters of water samples were identified based on the temperature and salinity. The two components (i.e. components 1 and 2) explained 100% of the point variability. Different water masses are indicated in green (EWM), grey (CWM), red (sOWM) and blue (ssOWM).
**Fig. S3.** Rarefaction curve of similarity‐based operational taxonomic units (OTUs) at 97% similarity level. (A) Viral *g23* and (B) bacterial 16S rRNA gene libraries from the nSCS.
**Fig. S4.** Frequency and abundance rank plot for viral (A‐B) and bacterial (C‐D) OTUs. A spline curve and scatter diagram were used to express the relationship between the frequency of OTUs occurrence and the number of OTUs and average contribution of OTUs to the community respectively. Each black dot represents an individual OTU.
**Fig. S5.** Bacterial community biodiversity and composition at the phylum (A) and class (B) level.
**Fig. S6.** SIMPER analysis of the dissimilarity among four viral oceanic water masses (cut‐off of low contribution: 90%). Only dominant *g23* OTUs were used.
**Table S1.** Location and environmental parameters of the 17 samples from the nSCS.
**Table S2.** Good's coverage, richness (Chao I) and diversity (Shannon and Simpson) indices across all samples at the 97% similarity level.
**Table S3.** Mantel test for the correlation between viral community composition and abiotic and abundance environmental variables using Pearson's coefficient. *P < 0.05, **P < 0.01 and ***P < 0.001.
**Table S4.** Mantel and partial Mantel tests for the correlation between the Exo T‐even viral community and other variables. ‘|’ indicates partial mantel test.
