# Supplementary material for: T4‐like myovirus community shaped by dispersal and deterministic processes in the South China Sea
Source: Environ Microbiol. 2020 Nov 3;23(2):1038–52. doi: 10.1111/1462-2920.15290 (PMC7984403; doi:10.1111/1462-2920.15290)
Supplement: Supplementary file 1 — Fig. S1. Location of the 11 sampling stations in the nSCS. Water samples were collected from surface and subsurface layers. Fig. S2. (A) Distribution of salinity and temperature among the 17 samples in the nSCS. (B) Three clusters of water samples were identified based on the temperature and salinity. The two components (i.e. components 1 and 2) explained 100% of the point variability. Different water masses are indicated in green (EWM), grey (CWM), red (sOWM) and blue (ssOWM). Fig. S3. Rarefaction curve of similarity‐based operational taxonomic units (OTUs) at 97% similarity level. (A) Viral g23 and (B) bacterial 16S rRNA gene libraries from the nSCS. Fig. S4. Frequency and abundance rank plot for viral (A‐B) and bacterial (C‐D) OTUs. A spline curve and scatter diagram were used to express the relationship between the frequency of OTUs occurrence and the number of OTUs and average contribution of OTUs to the community respectively. Each black dot represents an individual OTU. Fig. S5. Bacterial community biodiversity and composition at the phylum (A) and class (B) level. Fig. S6. SIMPER analysis of the dissimilarity among four viral oceanic water masses (cut‐off of low contribution: 90%). Only dominant g23 OTUs were used. Table S1. Location and environmental parameters of the 17 samples from the nSCS. Table S2. Good's coverage, richness (Chao I) and diversity (Shannon and Simpson) indices across all samples at the 97% similarity level. Table S3. Mantel test for the correlation between viral community composition and abiotic and abundance environmental variables using Pearson's coefficient. *P < 0.05, **P < 0.01 and ***P < 0.001. Table S4. Mantel and partial Mantel tests for the correlation between the Exo T‐even viral community and other variables. ‘|’ indicates partial mantel test. [file EMI-23-1038-s001.docx]

Supplementary Information for

**T4-like myovirus community shaped by dispersal and deterministic processes in the South China Sea**

Huifang Li^1^, Lu Liu^1^, Yu Wang^1^, Lanlan Cai^2^, Maoqiu He^3^, Long Wang^1^, Chen Hu^1^, Nianzhi Jiao^1^ and Rui Zhang^1^*

^1^ State Key Laboratory of Marine Environmental Science, College of Ocean and Earth Sciences, Fujian Key Laboratory of Marine Carbon Sequestration, Xiamen University, Xiang’an, Xiamen, Fujian 361102, People’s Republic of China

^2^ Department of Ocean Science, The Hong Kong University of Science and Technology, Hong Kong, China

^3^ State Key Laboratory of Trophic Oceanography, South China Sea Institute of Oceanology, Chinese Academy of Sciences, Guangzhou, China.

*Corresponding author: E-mail: [ruizhang@xmu.edu.cn](mailto:ruizhang@xmu.edu.cn); Tel: 86-592-2880152; Fax: +86-592-2185375;

This file includes:

Materials and Methods

Fig. S1-S6

Tables S1-S4

**PCR amplification, sequencing and phylogenetic analysis**

The *g23* fragments of the T4-type phages were amplified using the degenerate primers MZIA1 bis (5’-GATATTTGIGGIGTTCAGCCIATGA-3’) and MZIA6 (5’-CGCGGTTGATTTCCAGCATGATTTC-3’) as performed by File´e et al., (2005). The amplicons were sequenced using 454 GS FLX platform (Roche 454 Life Sciences, Branford, CT, USA). The sequences were quality screened with MOTHUR (v.1.41.1), and eliminated sequences that contained ambiguous bases >0, homopolymer length >8, and any sequences length <250bp (Schloss et al., 2009). The chimera sequences were removed using usearch61 method (Edgar, 2010). The most-dominant *g23* OTUs with a relative abundance >1% per sample (a total of 93 OTUs) were translated to their deduced amino acid sequences and aligned using MEGA7 (Kumar et al., 2016). Their closest culture relatives of the *g23* genes were examined using BLASTn within the NCBI website (Nucleotide collection (nt)). Phylogenetic tree of the *g23* genes and published *g23* sequence from environmental samples (open sea, estuary, paddy soil and lake) and isolated phages infecting *Synechococcus* and *Prochlorococcus* was constructed with maximum-likelihood method using RAxML, with 1,000 bootstraps (Stamatakis, 2014).

The conserved regions V3-V4 of the bacterial 16S rRNA genes were amplified using the primers 338F (5’-ACTCCTACGGGAGGCAGCAG-3’) and 806R (5’-GGACTACHVGGGTWTCTAAT-3’) (Mori et al., 2014; Li et al., 2018). Resulting amplicons were sequenced using the Illumina MiSeq PE300 platform (Illumina, San Diego, USA). The raw reads were trimmed with Trimmomatic and merged using FLASH (Magoc and Salzberg, 2011; Bolger et al., 2014) with the following criteria: (1) the reads were truncated at any site receiving an average quality score <20 over a 50 bp sliding window; (2) sequences whose overlap being longer than 10 bp were merged.

**Reference**

Bolger, A.M., Lohse, M., and Usadel, B. (2014) Trimmomatic: a flexible trimmer for Illumina sequence data. *Bioinformatics* 30: 2114-2120.

Edgar, R.C. (2010) Search and clustering orders of magnitude faster than BLAST. *Bioinformatics* 26: 2460-2461.

Filée, J., Tétart, F., Suttle, C.A., and Krisch, H. (2005) Marine T4-type bacteriophages, a ubiquitous component of the dark matter of the biosphere. *Proceedings of the National Academy of Sciences of the United States of America* 102: 12471-12476.

Li, Y., Sun, L.L., Sun, M.L., Su, H.N., Zhang, X.Y., Xie, B.B. et al. (2018) Vertical and horizontal biogeographic patterns and major factors affecting bacterial communities in the open South China Sea. *Scientific Reports* 8: 8800.

Magoc, T., and Salzberg, S.L. (2011) FLASH: fast length adjustment of short reads to improve genome assemblies. *Bioinformatics* 27: 2957-2963.

Mori, H., Maruyama, F., Kato, H., Toyoda, A., Dozono, A., Ohtsubo, Y. et al. (2014) Design and experimental application of a novel non-degenerate universal primer set that amplifies prokaryotic 16S rRNA genes with a low possibility to amplify eukaryotic rRNA genes. *DNA Research* 21: 217-227.

Kumar, S., Stecher, G., and Tamura, K. (2016) MEGA7: Molecular Evolutionary Genetics Analysis version 7.0 for bigger datasets. *Molecular Biology and Evolution* 33: 1870-1874.

Schloss, P.D., Westcott, S.L., Ryabin, T., Hall, J.R., Hartmann, M., Hollister, E.B. et al. (2009) Introducing mothur: open-source, platform-independent, community-supported software for describing and comparing microbial communities. *Applied and Environmental Microbiology* 75: 7537-7541.

Stamatakis, A. (2014) RAxML version 8: a tool for phylogenetic analysis and post-analysis of large phylogenies. *Bioinformatics* 30: 1312-1313.


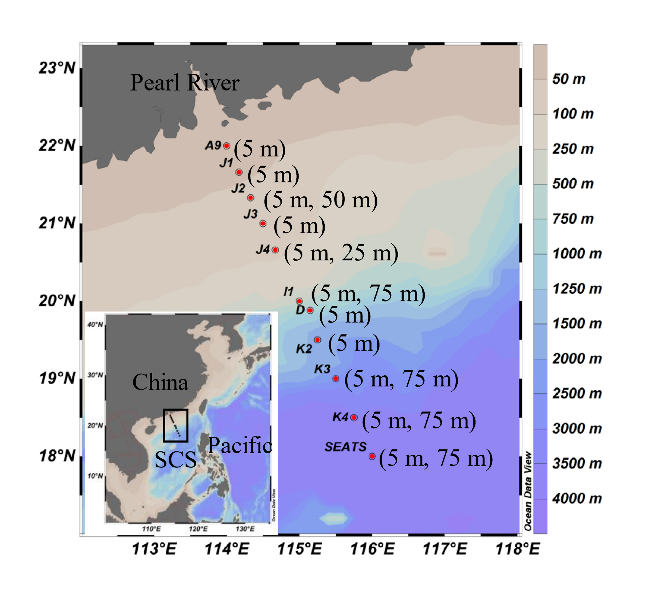


**Fig. S1** Location of the 11 sampling stations in the nSCS. Water samples were collected from surface and subsurface layers.


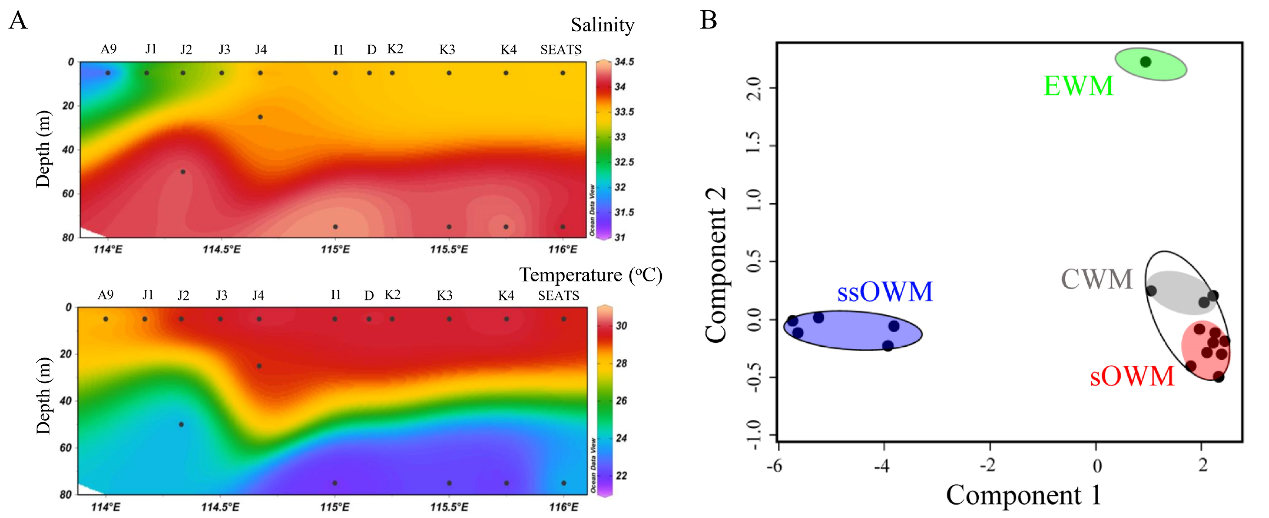


**Fig. S2** (A) Distribution of salinity and temperature among the 17 samples in the nSCS. (B) Three clusters of water samples were identified based on the temperature and salinity. The two components (i.e., components 1 and 2) explained 100% of the point variability. Different water masses are indicated in green (EWM), gray (CWM), red (sOWM) and blue (ssOWM).


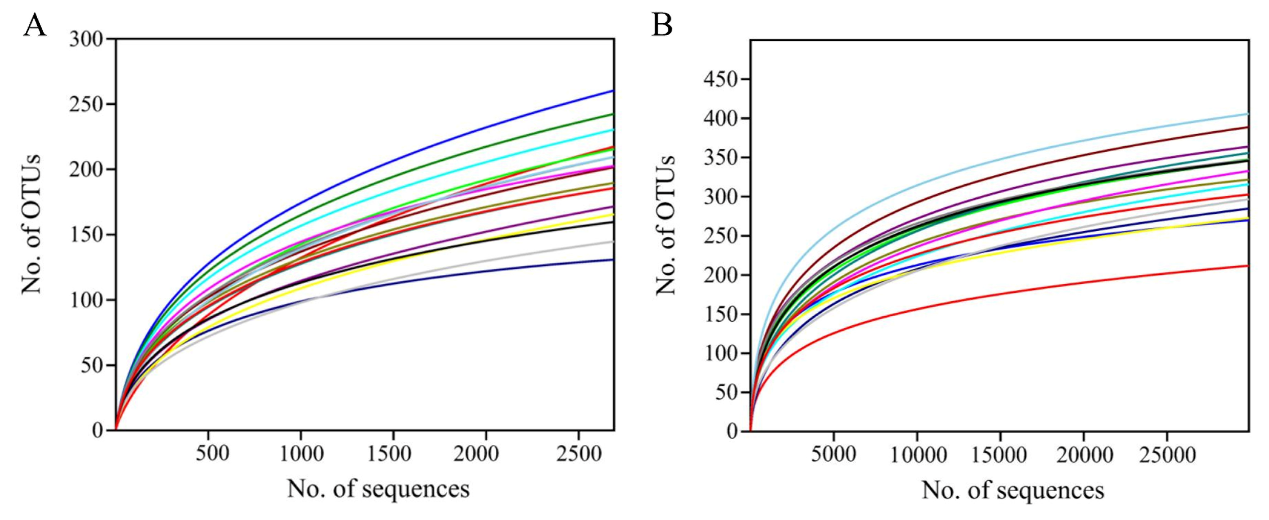


**Fig. S3** Rarefaction curve of similarity-based operational taxonomic units (OTUs) at 97% similarity level. (A) Viral *g23* and (B) bacterial 16S rRNA gene libraries from the nSCS.


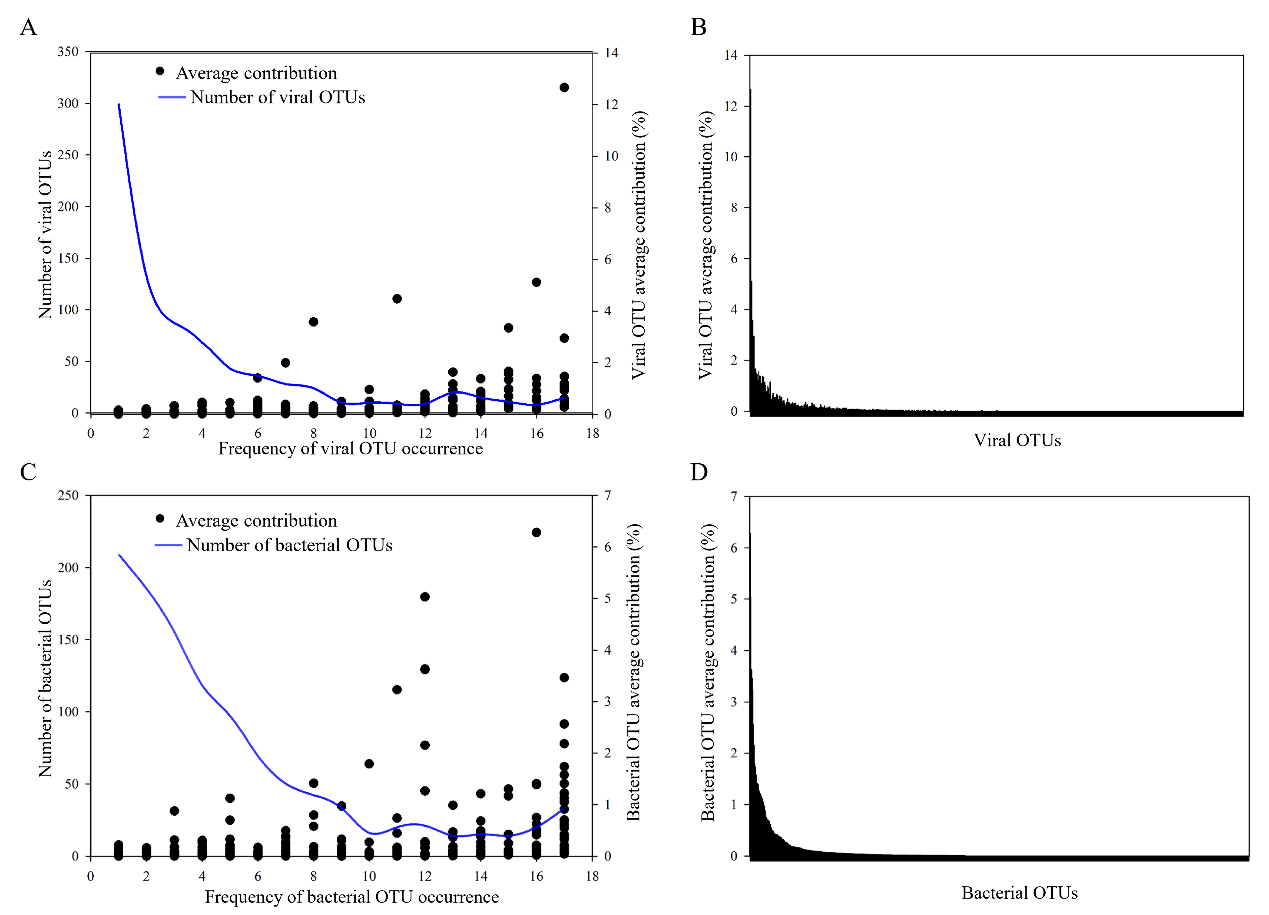


**Fig. S4** Frequency and abundance rank plot for viral (A-B) and bacterial (C-D) OTUs. A spline curve and scatter diagram were used to express the relationship between the frequency of OTUs occurrence and the number of OTUs and average contribution of OTUs to the community, respectively. Each black dot represents an individual OTU.


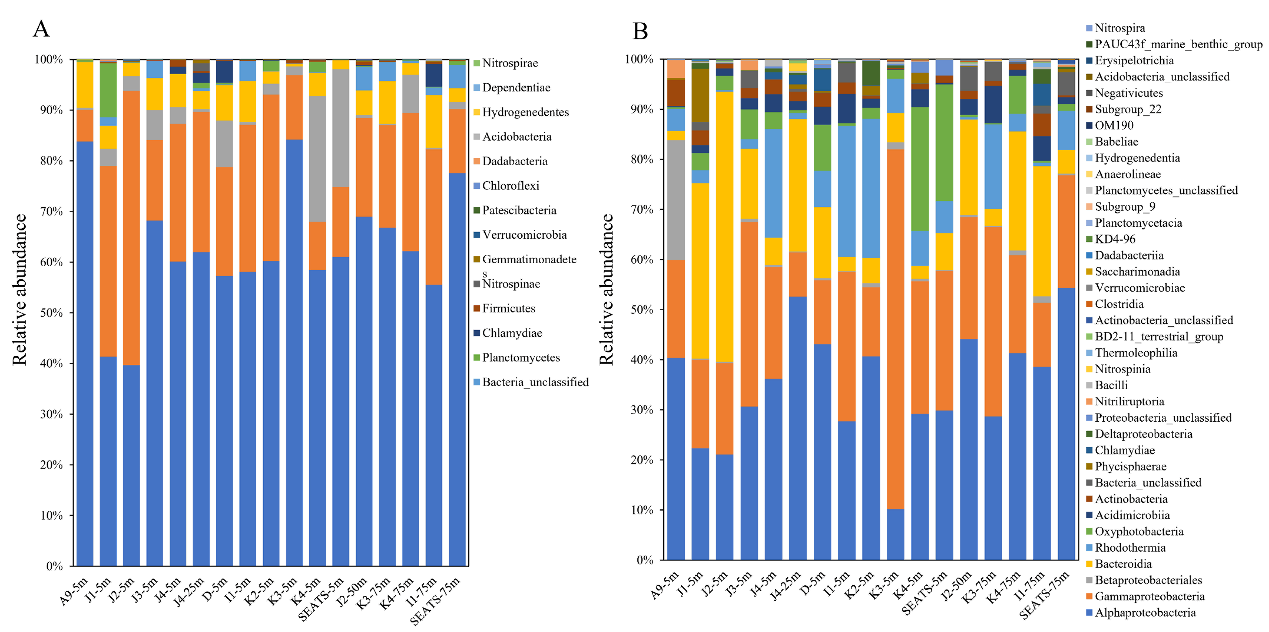


**Fig. S5** Bacterial community biodiversity and composition at the phylum (A) and class (B)level.


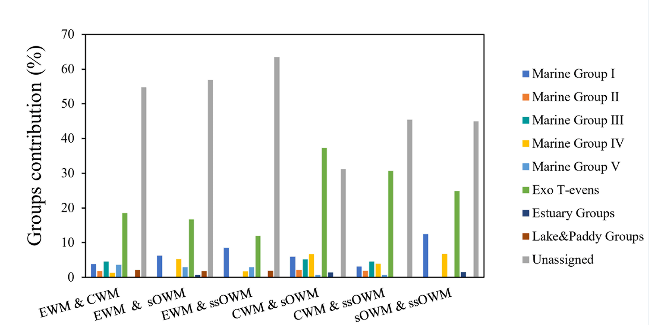


**Fig. S6** SIMPER analysis of the dissimilarity among four viral oceanic water masses (cut off of low contribution: 90%). Only dominant *g23* OTUs were used.

**Table S1** Location and environmental parameters of the 17 samples from the nSCS.

| **Sample** | **Depth (m)** | **Longitude (^o^E)** | **Latitude (^o^N)** | **Salinity** | **Temperature (^o^C)** | **NO_3_^-^+NO_2_^-^(μmol/L)** | **SiO_3_^2-^**  **(μmol/L)** | **PO_4_^3-^**  **(μmol/L)** | **HB^a^ (cell/mL)** | **Pro^a^ (cell/mL)** | **Syn^a^ (cell/mL)** | **Euk^a^ (cell/mL)** |
| --- | --- | --- | --- | --- | --- | --- | --- | --- | --- | --- | --- | --- |
| A9-5m | 5 | 114.00 | 22.00 | 31.19 | 28.1 | 59.285 | 56.242 | 0.400 | 3.16E+06 | 3.03E+04 | 9.36E+02 | 3.00E+03 |
| J1-5m | 5 | 114.17 | 21.66 | 33.12 | 28.6 | 30.302 | 69.389 | 2.138 | 6.71E+05 | 2.34E+03 | 7.56E+02 | 8.04E+02 |
| J2-5m | 5 | 114.33 | 21.33 | 33.04 | 29.7 | 0.085 | 0.630 | 0.183 | 1.11E+06 | 2.56E+04 | 3.90E+03 | 1.74E+02 |
| J3-5m | 5 | 114.50 | 21.00 | 33.08 | 29.6 | 0.055 | 0.795 | 0.191 | 1.26E+06 | 4.11E+04 | 4.90E+03 | 2.30E+02 |
| J4-5m | 5 | 114.67 | 20.66 | 33.70 | 30.0 | 0.093 | 0.254 | 0.173 | 9.80E+05 | 4.23E+04 | 5.40E+02 | 4.50E+02 |
| J4-25m | 25 | 114.67 | 20.66 | 33.68 | 29.4 | -- | -- | -- | 1.45E+06 | 2.28E+05 | 1.14E+04 | 3.29E+03 |
| I1-5m | 5 | 115.00 | 20.00 | 33.53 | 29.7 | 0.139 | 0.912 | 6.950 | 8.95E+05 | 3.79E+04 | 2.83E+03 | 4.38E+02 |
| D-5m | 5 | 115.15 | 19.88 | 33.51 | 29.9 | 0.087 | 0.956 | 0.160 | 1.12E+06 | 2.08E+04 | 5.76E+03 | 8.25E+02 |
| K2-5m | 5 | 115.25 | 19.50 | 33.44 | 29.8 | 0.079 | 1.065 | 6.757 | 8.36E+05 | 3.86E+04 | 3.09E+03 | 6.69E+02 |
| K3-5m | 5 | 115.50 | 19.00 | 33.37 | 29.8 | 0.058 | 1.288 | 0.190 | 9.58E+05 | 1.44E+05 | 4.55E+03 | 5.39E+02 |
| K4-5m | 5 | 115.75 | 18.50 | 33.40 | 29.9 | 0.155 | 0.947 | 0.247 | 8.04E+05 | 3.51E+04 | 2.60E+03 | 4.21E+02 |
| SEATS-5m | 5 | 116.00 | 18.00 | 33.35 | 29.5 | 0.127 | 0.748 | 0.203 | 1.10E+06 | 6.41E+03 | 3.24E+02 | 1.20E+02 |
| J2-50m | 50 | 114.33 | 21.33 | 34.25 | 23.7 | 0.071 | 0.809 | 0.203 | 8.74E+05 | 8.83E+03 | 2.80E+03 | 2.45E+03 |
| I1-75m | 75 | 115.00 | 20.00 | 34.36 | 21.9 | 6.014 | 15.983 | 0.544 | 6.15E+05 | 7.63E+04 | 2.31E+04 | 2.02E+04 |
| K3-75m | 75 | 115.50 | 19.00 | 34.22 | 22.3 | 1.593 | 2.075 | 0.357 | 4.93E+05 | 5.33E+04 | 1.08E+03 | 3.20E+03 |
| K4-75m | 75 | 115.75 | 18.50 | 34.32 | 21.9 | 1.950 | 3.277 | 0.389 | 7.98E+05 | 3.47E+04 | 7.67E+02 | 1.71E+03 |
| SEATS-75m | 75 | 116.00 | 18.00 | 34.08 | 23.8 | 2.481 | 3.638 | 0.420 | 6.62E+05 | 1.02E+04 | 7.20E+01 | 2.70E+02 |

Abbreviations: HB^a^, Heterotrophic bacteria abundance; Pro^a^, *Prochlorococcus* abundance; Syn^a^, *Synechococcus* abundance; Euk^a^, Picoeukaryotes abundance.

**Table S2** Good’s coverage, richness (Chao I) and diversity (Shannon and Simpson) indices across all samples at the 97% similarity level.

|  | Sample | Chao I | Coverage | Shannon | Simpson |
| --- | --- | --- | --- | --- | --- |
| Virus | A9-5m | 334 | 0.963 | 3.679 | 0.679 |
|  | J1-5m | 389 | 0.963 | 6.334 | 0.974 |
|  | J2-5m | 341 | 0.968 | 6.157 | 0.968 |
|  | J3-5m | 371 | 0.967 | 5.959 | 0.959 |
|  | J4-5m | 279 | 0.975 | 5.217 | 0.936 |
|  | J4-25m | 314 | 0.970 | 5.707 | 0.959 |
|  | I1-5m | 253 | 0.977 | 5.658 | 0.959 |
|  | D-5m | 284 | 0.973 | 5.607 | 0.938 |
|  | K2-5m | 271 | 0.977 | 5.864 | 0.960 |
|  | K3-5m | 145 | 0.990 | 4.957 | 0.927 |
|  | K4-5m | 240 | 0.977 | 5.135 | 0.897 |
|  | SEATS-5m | 308 | 0.974 | 4.614 | 0.887 |
|  | J2-50m | 315 | 0.970 | 5.758 | 0.950 |
|  | I1-75m | 280 | 0.972 | 5.403 | 0.940 |
|  | K3-75m | 190 | 0.982 | 4.678 | 0.903 |
|  | K4-75m | 200 | 0.982 | 5.265 | 0.940 |
|  | SEATS-75m | 243 | 0.978 | 5.607 | 0.958 |
| Bacteria | A9-5m | 265 | 0.998 | 4.274 | 0.903 |
|  | J1-5m | 323 | 0.998 | 5.062 | 0.930 |
|  | J2-5m | 443 | 0.997 | 5.047 | 0.930 |
|  | J3-5m | 393 | 0.997 | 5.142 | 0.942 |
|  | J4-5m | 430 | 0.997 | 5.603 | 0.945 |
|  | J4-25m | 451 | 0.997 | 5.016 | 0.924 |
|  | I1-5m | 368 | 0.998 | 4.785 | 0.906 |
|  | D-5m | 497 | 0.997 | 5.724 | 0.962 |
|  | K2-5m | 428 | 0.997 | 5.049 | 0.937 |
|  | K3-5m | 399 | 0.997 | 3.105 | 0.641 |
|  | K4-5m | 435 | 0.997 | 4.829 | 0.911 |
|  | SEATS-5m | 371 | 0.998 | 5.004 | 0.925 |
|  | J2-50m | 400 | 0.998 | 5.311 | 0.934 |
|  | I1-75m | 522 | 0.997 | 5.654 | 0.934 |
|  | K3-75m | 399 | 0.997 | 4.557 | 0.911 |
|  | K4-75m | 412 | 0.998 | 4.975 | 0.916 |
|  | SEATS-75m | 378 | 0.998 | 4.540 | 0.847 |

**Table S3** Mantel test for the correlation between viral community composition and abiotic and abundance environmental variables using Pearson’s coefficient. **P* < 0.05, ***P* < 0.01 and ****P* < 0.001.

| Variables | T4-like virus | CWM | sOWM | ssOWM |
| --- | --- | --- | --- | --- |
| Temperature | 0.4463** | 0.4331 | -0.0633 | 0.1346 |
| Salinity | 0.7447** | -0.4868 | 0.3062 | -0.1828 |
| NO_3_^-^ + NO_2_^-^ | 0.5408** | 0.5138 | 0.1348 | 0.0786 |
| PO_4_^3-^ | -0.1788 | 0.5100 | 0.0329 | 0.5714 |
| SiO_3_^2-^ | 0.3983* | 0.5113 | 0.5468 | -0.2013 |
| HB^a^ | 0.5878** | 0.7717 | -0.2611 | 0.3256 |
| Pro^a^ | -0.083 | 0.9497 | 0.2659 | -0.0724 |
| Syn^a^ | -0.03844 | 0.7548 | 0.0645 | -0.2770 |
| Euk^a^ | -0.04038 | 0.4351 | -0.2818 | -0.3204 |

**Table S4** Mantel and partial Mantel tests for the correlation between Exo T-evens viral community and other variables. “|” indicates partial mantel test.

| Variables | r | *P* |
| --- | --- | --- |
| Abiotic and abundance | 0.4370 | 0.001 |
| Abiotic and abundance \| bacterial community | 0.3096 | 0.043 |
| Abiotic and abundance \| spatial variables | 0.3851 | 0.011 |
| Bacterial community | 0.5239 | 0.001 |
| Bacterial community \| abiotic and abundance | 0.4350 | 0.001 |
| Bacterial community \| spatial variables | 0.4384 | 0.001 |
| Spatial variables | 0.3687 | 0.002 |
| Spatial variables \| abiotic and abundance | 0.3007 | 0.008 |
| Spatial variables \| bacterial community | 0.1945 | 0.028 |
